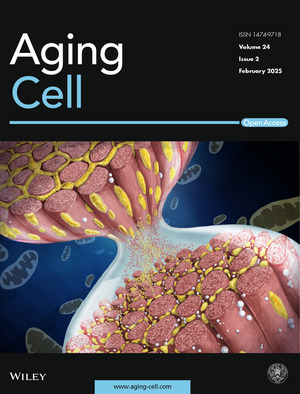# Featured Cover

**DOI:** 10.1111/acel.70017

**Published:** 2025-02-13

**Authors:** Richie P. Goulding, Braeden T. Charlton, Ellen A. Breedveld, Matthijs van der Laan, Anne R. Strating, Wendy Noort, Aryna Kolodyazhna, Brent Appelman, Michèle van Vugt, Anita E. Grootemaat, Nicole N. van der Wel, Jos J. de Koning, Frank W. Bloemers, Rob C. I. Wüst

## Abstract

Cover legend: The cover image is based on the article Skeletal muscle mitochondrial fragmentation predicts age‐associated decline in physical capacity by Rob Wüst *et al*., https://doi.org/10.1111/acel.14386.